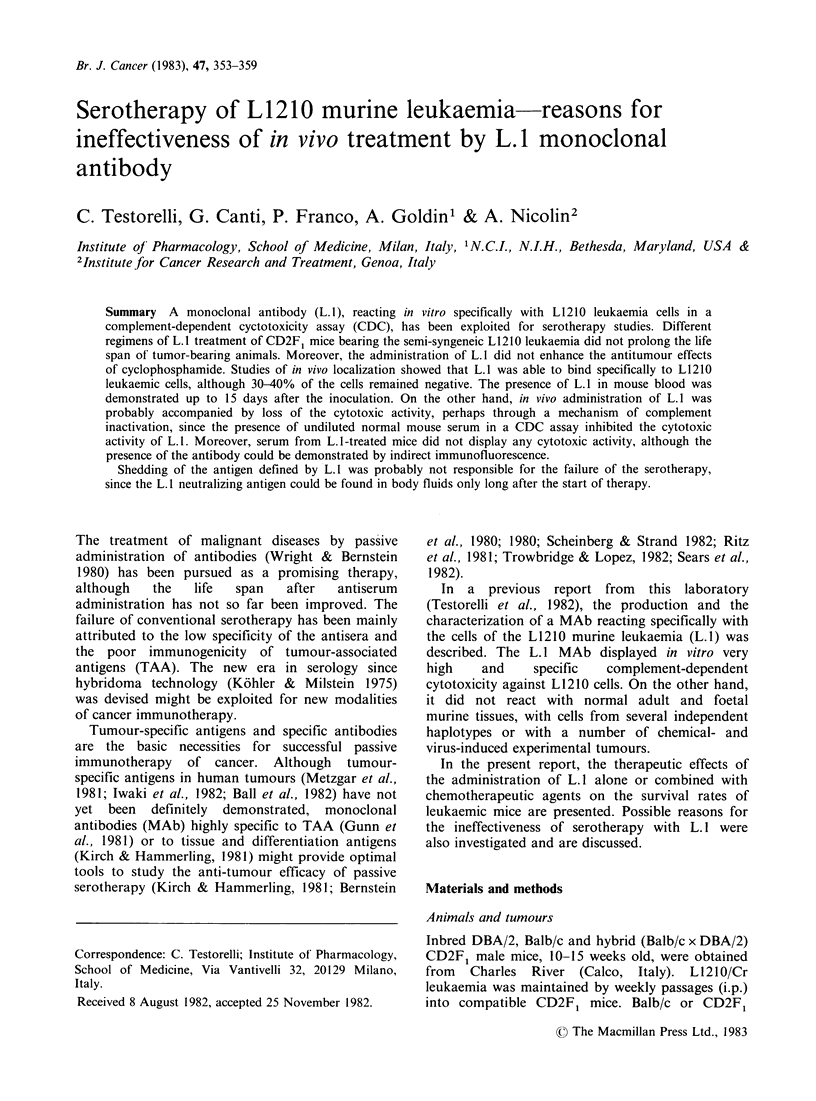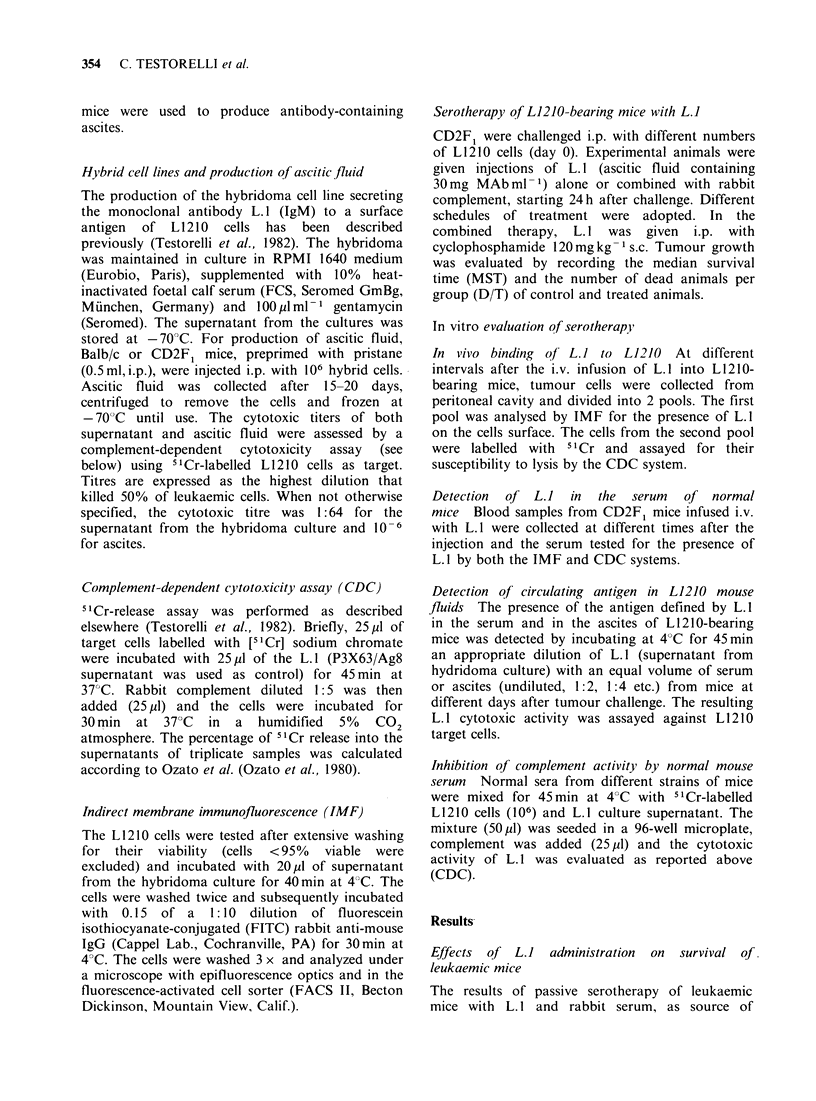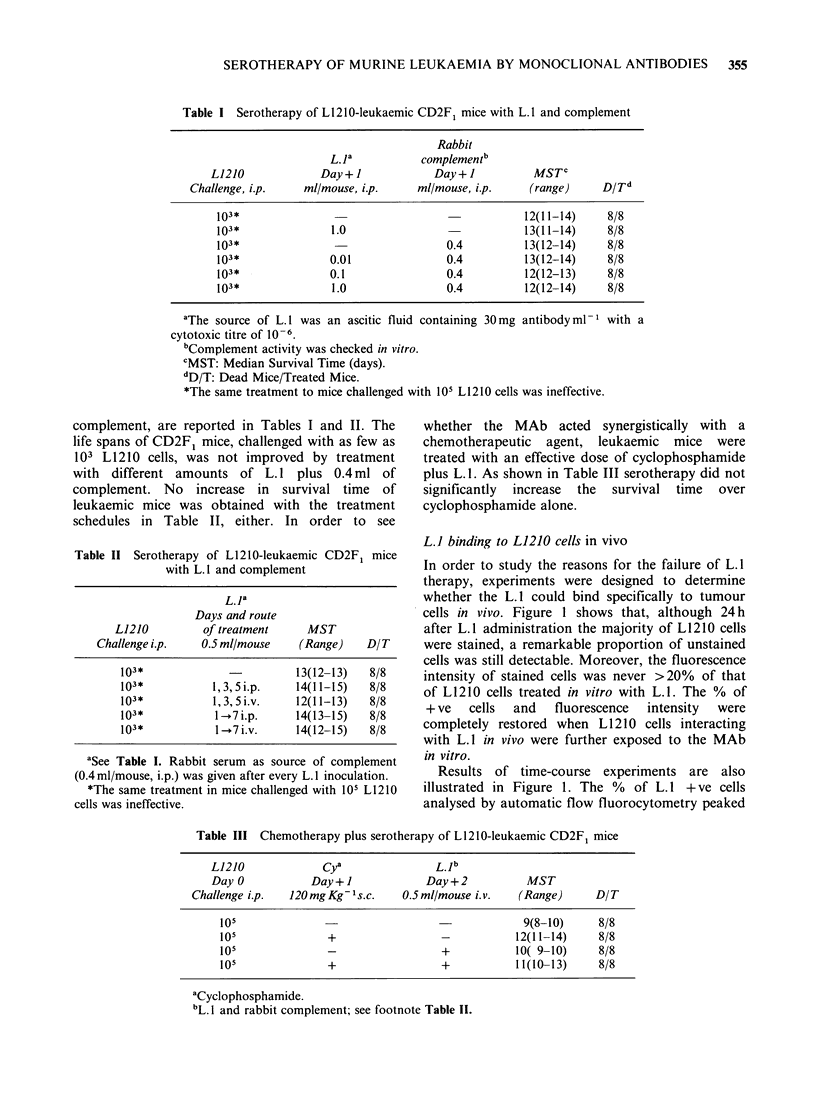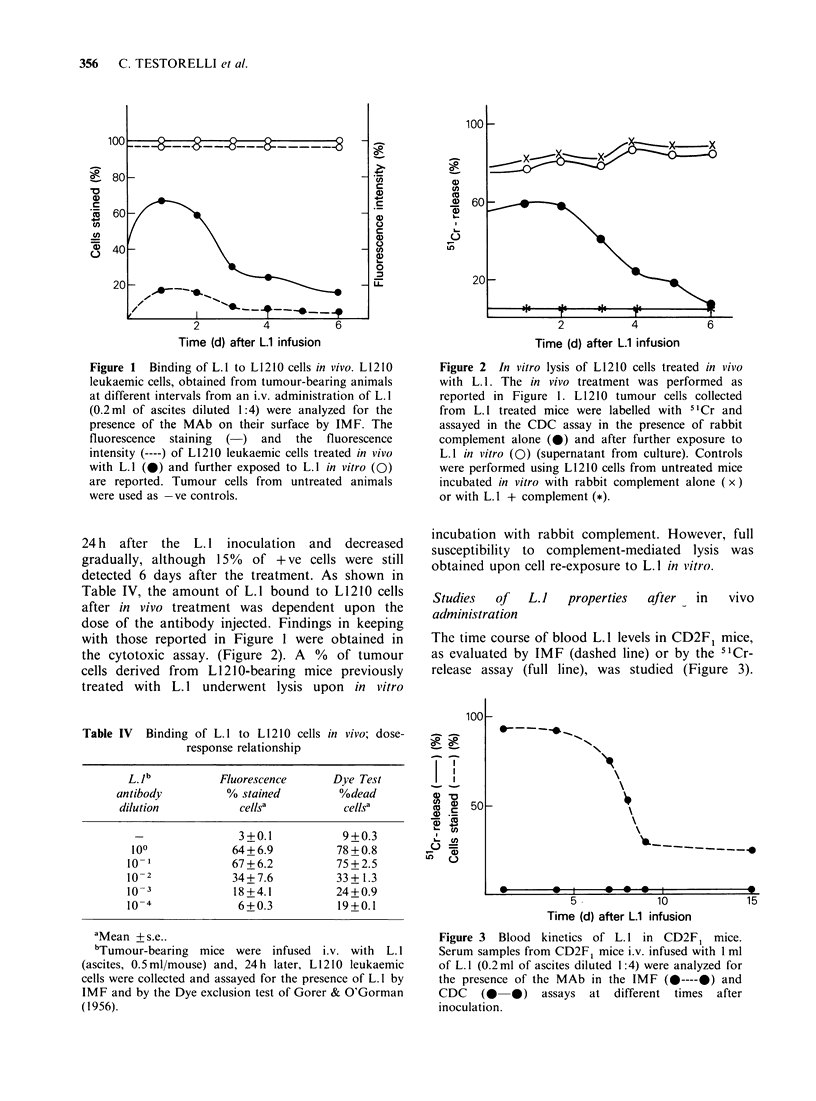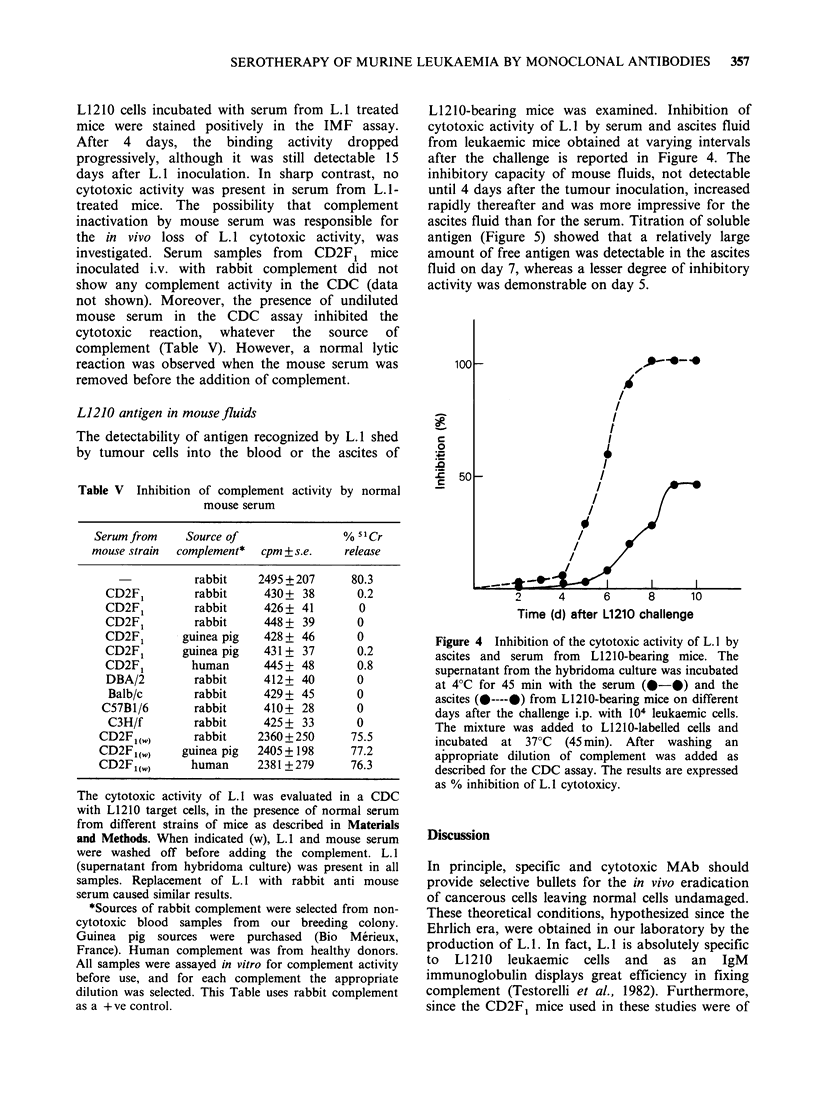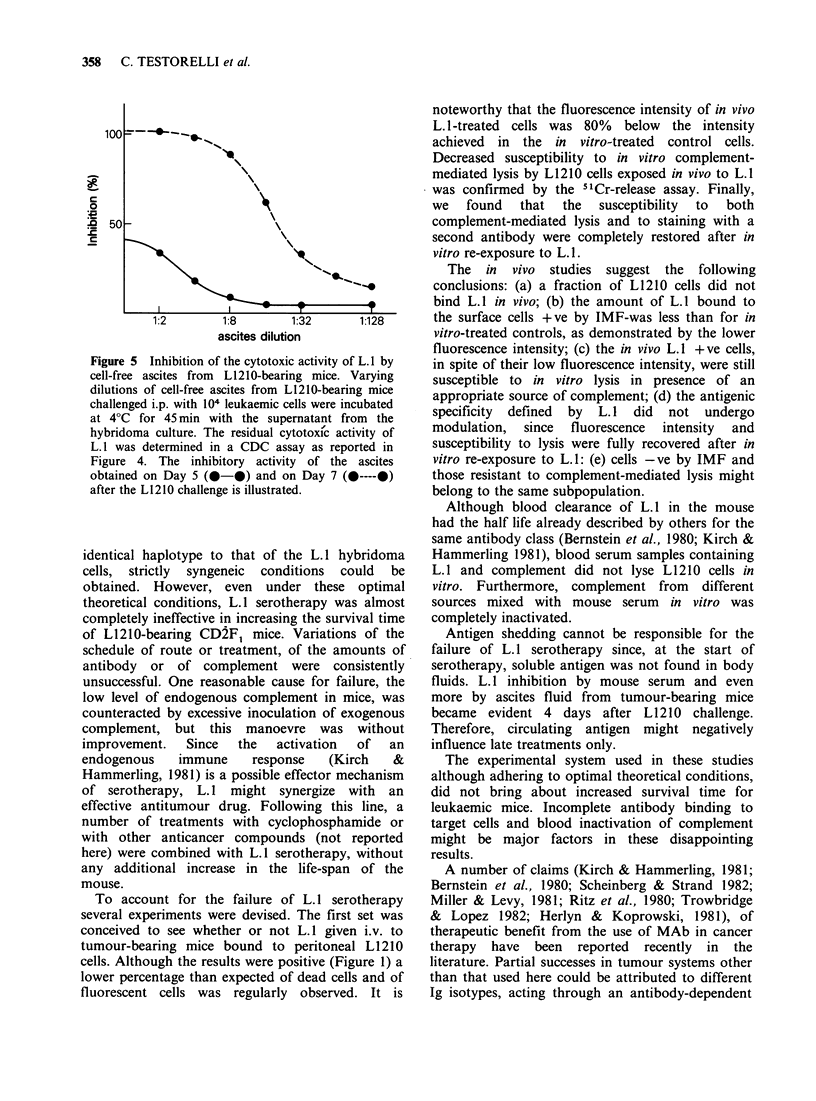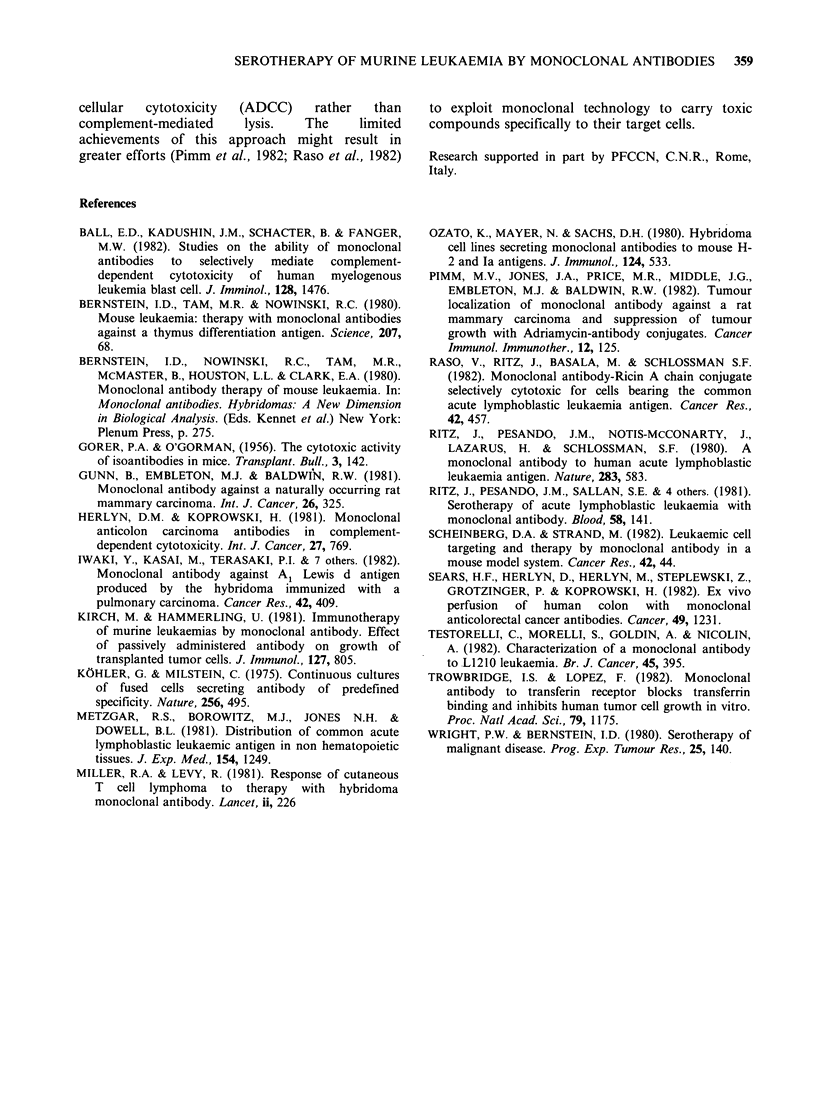# Serotherapy of L1210 murine leukaemia--reasons for ineffectiveness of in vivo treatment by L.1 monoclonal antibody.

**DOI:** 10.1038/bjc.1983.53

**Published:** 1983-03

**Authors:** C. Testorelli, G. Canti, P. Franco, A. Goldin, A. Nicolin

## Abstract

A monoclonal antibody (L.1), reacting in vitro specifically with L1210 leukaemia cells in a complement-dependent cytotoxicity assay (CDC), has been exploited for serotherapy studies. Different regiments of L.1 treatment of CD2F1 mice bearing the semi-syngeneic L1210 leukaemia did not prolong the life span of tumor-bearing animals. Moreover, the administration of L.1 did not enhance the antitumour effects of cyclophosphamide. Studies of in vivo localization showed that L.1 was able to bind specifically to L1210 leukaemic cells, although 30-40% of the cells remained negative. The presence of L.1 in mouse blood was demonstrated up to 15 days after the inoculation. On the other hand, in vivo administration of L.1 was probably accompanied by loss of the cytotoxic activity, perhaps through a mechanism of complement inactivation, since the presence of undiluted normal mouse serum in a CDC assay inhibited the cytotoxic activity of L.1. Moreover, serum from L.1-treated mice did not display any cytotoxic activity, although the presence of the antibody could be demonstrated by indirect immunofluorescence. Shedding of the antigen defined by L.1 was probably not responsible for the failure of the serotherapy, since the L.1 neutralizing antigen could be found in body fluids only long after the start of therapy.


*Br. J. Cancer* (1983), **47**, 353–359

### Serotherapy of L1210 murine leukaemia—reasons for ineffectiveness of *in vivo* treatment by L.1 monoclonal antibody

C. Testorelli, G. Canti, P. Franco, A. Goldin[1] & A. Nicolin[2]

*Institute of Pharmacology, School of Medicine, Milan, Italy, [1]N.C.I., N.I.H., Bethesda, Maryland, USA & [2]Institute for Cancer Research and Treatment, Genoa, Italy*

**Summary** A monoclonal antibody (L.1), reacting *in vitro* specifically with L1210 leukaemia cells in a complement-dependent cyctotoxicity assay (CDC), has been exploited for serotherapy studies. Different regimens of L.1 treatment of $CD2F_1$ mice bearing the semi-syngeneic L1210 leukaemia did not prolong the life span of tumor-bearing animals. Moreover, the administration of L.1 did not enhance the antitumour effects of cyclophosphamide. Studies of *in vivo* localization showed that L.1 was able to bind specifically to L1210 leukaemic cells, although 30–40% of the cells remained negative. The presence of L.1 in mouse blood was demonstrated up to 15 days after the inoculation. On the other hand, *in vivo* administration of L.1 was probably accompanied by loss of the cytotoxic activity, perhaps through a mechanism of complement inactivation, since the presence of undiluted normal mouse serum in a CDC assay inhibited the cytotoxic activity of L.1. Moreover, serum from L.1-treated mice did not display any cytotoxic activity, although the presence of the antibody could be demonstrated by indirect immunofluorescence.

Shedding of the antigen defined by L.1 was probably not responsible for the failure of the serotherapy, since the L.1 neutralizing antigen could be found in body fluids only long after the start of therapy.

The treatment of malignant diseases by passive administration of antibodies (Wright & Bernstein 1980) has been pursued as a promising therapy, although the life span after antiserum administration has not so far been improved. The failure of conventional serotherapy has been mainly attributed to the low specificity of the antisera and the poor immunogenicity of tumour-associated antigens (TAA). The new era in serology since hybridoma technology (Köhler & Milstein 1975) was devised might be exploited for new modalities of cancer immunotherapy.

Tumour-specific antigens and specific antibodies are the basic necessities for successful passive immunotherapy of cancer. Although tumour-specific antigens in human tumours (Metzgar *et al.*, 1981; Iwaki *et al.*, 1982; Ball *et al.*, 1982) have not yet been definitely demonstrated, monoclonal antibodies (MAb) highly specific to TAA (Gunn *et al.*, 1981) or to tissue and differentiation antigens (Kirch & Hammerling, 1981) might provide optimal tools to study the anti-tumour efficacy of passive serotherapy (Kirch & Hammerling, 1981; Bernstein *et al.*, 1980; 1980; Scheinberg & Strand 1982; Ritz *et al.*, 1981; Trowbridge & Lopez, 1982; Sears *et al.*, 1982).

In a previous report from this laboratory (Testorelli *et al.*, 1982), the production and the characterization of a MAb reacting specifically with the cells of the L1210 murine leukaemia (L.1) was described. The L.1 MAb displayed *in vitro* very high and specific complement-dependent cytotoxicity against L1210 cells. On the other hand, it did not react with normal adult and foetal murine tissues, with cells from several independent haplotypes or with a number of chemical- and virus-induced experimental tumours.

In the present report, the therapeutic effects of the administration of L.1 alone or combined with chemotherapeutic agents on the survival rates of leukaemic mice are presented. Possible reasons for the ineffectiveness of serotherapy with L.1 were also investigated and are discussed.

### Materials and methods

### *Animals and tumours*

Inbred DBA/2, Balb/c and hybrid (Balb/c × DBA/2) $CD2F_1$ male mice, 10–15 weeks old, were obtained from Charles River (Calco, Italy). L1210/Cr leukaemia was maintained by weekly passages (i.p.) into compatible $CD2F_1$ mice. Balb/c or $CD2F_1$

Correspondence: C. Testorelli; Institute of Pharmacology, School of Medicine, Via Vantivelli 32, 20129 Milano, Italy.

Received 8 August 1982, accepted 25 November 1982.

© The Macmillan Press Ltd., 1983

mice were used to produce antibody-containing ascites.

*Hybrid cell lines and production of ascitic fluid*

The production of the hybridoma cell line secreting the monoclonal antibody L.1 (IgM) to a surface antigen of L1210 cells has been described previously (Testorelli *et al.*, 1982). The hybridoma was maintained in culture in RPMI 1640 medium (Eurobio, Paris), supplemented with 10% heat-inactivated foetal calf serum (FCS, Seromed GmBg, München, Germany) and 100 $\mu l\, ml^{-1}$ gentamycin (Seromed). The supernatant from the cultures was stored at −70°C. For production of ascitic fluid, Balb/c or $CD2F_1$ mice, preprimed with pristane (0.5 ml, i.p.), were injected i.p. with $10^6$ hybrid cells. Ascitic fluid was collected after 15–20 days, centrifuged to remove the cells and frozen at −70°C until use. The cytotoxic titers of both supernatant and ascitic fluid were assessed by a complement-dependent cytotoxicity assay (see below) using $^{51}Cr$-labelled L1210 cells as target. Titres are expressed as the highest dilution that killed 50% of leukaemic cells. When not otherwise specified, the cytotoxic titre was 1:64 for the supernatant from the hybridoma culture and $10^{-6}$ for ascites.

*Complement-dependent cytotoxicity assay (CDC)*

$^{51}Cr$-release assay was performed as described elsewhere (Testorelli *et al.*, 1982). Briefly, 25 $\mu l$ of target cells labelled with [$^{51}Cr$] sodium chromate were incubated with 25 $\mu l$ of the L.1 (P3X63/Ag8 supernatant was used as control) for 45 min at 37°C. Rabbit complement diluted 1:5 was then added (25 $\mu l$) and the cells were incubated for 30 min at 37°C in a humidified 5% $CO_2$ atmosphere. The percentage of $^{51}Cr$ release into the supernatants of triplicate samples was calculated according to Ozato *et al.* (Ozato *et al.*, 1980).

*Indirect membrane immunofluorescence (IMF)*

The L1210 cells were tested after extensive washing for their viability (cells <95% viable were excluded) and incubated with 20 $\mu l$ of supernatant from the hybridoma culture for 40 min at 4°C. The cells were washed twice and subsequently incubated with 0.15 of a 1:10 dilution of fluorescein isothiocyanate-conjugated (FITC) rabbit anti-mouse IgG (Cappel Lab., Cochranville, PA) for 30 min at 4°C. The cells were washed 3× and analyzed under a microscope with epifluorescence optics and in the fluorescence-activated cell sorter (FACS II, Becton Dickinson, Mountain View, Calif.).

*Serotherapy of L1210-bearing mice with L.1*

$CD2F_1$ were challenged i.p. with different numbers of L1210 cells (day 0). Experimental animals were given injections of L.1 (ascitic fluid containing 30 mg MAb $ml^{-1}$) alone or combined with rabbit complement, starting 24 h after challenge. Different schedules of treatment were adopted. In the combined therapy, L.1 was given i.p. with cyclophosphamide 120 mg $kg^{-1}$ s.c. Tumour growth was evaluated by recording the median survival time (MST) and the number of dead animals per group (D/T) of control and treated animals.

In vitro *evaluation of serotherapy*

*In vivo binding of L.1 to L1210* At different intervals after the i.v. infusion of L.1 into L1210-bearing mice, tumour cells were collected from peritoneal cavity and divided into 2 pools. The first pool was analysed by IMF for the presence of L.1 on the cells surface. The cells from the second pool were labelled with $^{51}Cr$ and assayed for their susceptibility to lysis by the CDC system.

*Detection of L.1 in the serum of normal mice* Blood samples from $CD2F_1$ mice infused i.v. with L.1 were collected at different times after the injection and the serum tested for the presence of L.1 by both the IMF and CDC systems.

*Detection of circulating antigen in L1210 mouse fluids* The presence of the antigen defined by L.1 in the serum and in the ascites of L1210-bearing mice was detected by incubating at 4°C for 45 min an appropriate dilution of L.1 (supernatant from hydridoma culture) with an equal volume of serum or ascites (undiluted, 1:2, 1:4 etc.) from mice at different days after tumour challenge. The resulting L.1 cytotoxic activity was assayed against L1210 target cells.

*Inhibition of complement activity by normal mouse serum* Normal sera from different strains of mice were mixed for 45 min at 4°C with $^{51}Cr$-labelled L1210 cells ($10^6$) and L.1 culture supernatant. The mixture (50 $\mu l$) was seeded in a 96-well microplate, complement was added (25 $\mu l$) and the cytotoxic activity of L.1 was evaluated as reported above (CDC).

### Results

*Effects of L.1 administration on survival of leukaemic mice*

The results of passive serotherapy of leukaemic mice with L.1 and rabbit serum, as source of

**Table I** Serotherapy of L1210-leukaemic $CD2F_1$ mice with L.1 and complement

| *L1210 Challenge, i.p.* | *L.1*[a] *Day+1 ml/mouse, i.p.* | *Rabbit complement*[b] *Day+1 ml/mouse, i.p.* | *MST*[c] *(range)* | *D/T*[d] |
|---|---|---|---|---|
| $10^3$* | — | — | 12(11–14) | 8/8 |
| $10^3$* | 1.0 | — | 13(11–14) | 8/8 |
| $10^3$* | — | 0.4 | 13(12–14) | 8/8 |
| $10^3$* | 0.01 | 0.4 | 13(12–14) | 8/8 |
| $10^3$* | 0.1 | 0.4 | 12(12–13) | 8/8 |
| $10^3$* | 1.0 | 0.4 | 12(12–14) | 8/8 |

[a]The source of L.1 was an ascitic fluid containing 30 mg antibody $ml^{-1}$ with a cytotoxic titre of $10^{-6}$.
[b]Complement activity was checked *in vitro*.
[c]MST: Median Survival Time (days).
[d]D/T: Dead Mice/Treated Mice.
*The same treatment to mice challenged with $10^5$ L1210 cells was ineffective.

complement, are reported in Tables I and II. The life spans of $CD2F_1$ mice, challenged with as few as $10^3$ L1210 cells, was not improved by treatment with different amounts of L.1 plus 0.4 ml of complement. No increase in survival time of leukaemic mice was obtained with the treatment schedules in Table II, either. In order to see whether the MAb acted synergistically with a chemotherapeutic agent, leukaemic mice were treated with an effective dose of cyclophosphamide plus L.1. As shown in Table III serotherapy did not significantly increase the survival time over cyclophosphamide alone.

**Table II** Serotherapy of L1210-leukaemic $CD2F_1$ mice with L.1 and complement

| *L1210 Challenge i.p.* | *L.1*[a] *Days and route of treatment 0.5 ml/mouse* | *MST (Range)* | *D/T* |
|---|---|---|---|
| $10^3$* | — | 13(12–13) | 8/8 |
| $10^3$* | 1, 3, 5 i.p. | 14(11–15) | 8/8 |
| $10^3$* | 1, 3, 5 i.v. | 12(11–13) | 8/8 |
| $10^3$* | 1→7 i.p. | 14(13–15) | 8/8 |
| $10^3$* | 1→7 i.v. | 14(12–15) | 8/8 |

[a]See **Table I.** Rabbit serum as source of complement (0.4 ml/mouse, i.p.) was given after every L.1 inoculation.
*The same treatment in mice challenged with $10^5$ L1210 cells was ineffective.

### *L.1 binding to L1210 cells* in vivo

In order to study the reasons for the failure of L.1 therapy, experiments were designed to determine whether the L.1 could bind specifically to tumour cells *in vivo*. Figure 1 shows that, although 24 h after L.1 administration the majority of L1210 cells were stained, a remarkable proportion of unstained cells was still detectable. Moreover, the fluorescence intensity of stained cells was never >20% of that of L1210 cells treated *in vitro* with L.1. The % of +ve cells and fluorescence intensity were completely restored when L1210 cells interacting with L.1 *in vivo* were further exposed to the MAb *in vitro*.

Results of time-course experiments are also illustrated in Figure 1. The % of L.1 +ve cells analysed by automatic flow fluorocytometry peaked

**Table III** Chemotherapy plus serotherapy of L1210-leukaemic $CD2F_1$ mice

| *L1210 Day 0 Challenge i.p.* | *Cy*[a] *Day+1 120 mg $Kg^{-1}$ s.c.* | *L.1*[b] *Day+2 0.5 ml/mouse i.v.* | *MST (Range)* | *D/T* |
|---|---|---|---|---|
| $10^5$ | — | — | 9(8–10) | 8/8 |
| $10^5$ | + | – | 12(11–14) | 8/8 |
| $10^5$ | – | + | 10( 9–10) | 8/8 |
| $10^5$ | + | + | 11(10–13) | 8/8 |

[a]Cyclophosphamide.
[b]L.1 and rabbit complement; see footnote **Table II.**

**Figure 1** Binding of L.1 to L1210 cells *in vivo*. L1210 leukaemic cells, obtained from tumour-bearing animals at different intervals from an i.v. administration of L.1 (0.2 ml of ascites diluted 1:4) were analyzed for the presence of the MAb on their surface by IMF. The fluorescence staining (—) and the fluorescence intensity (----) of L1210 leukaemic cells treated *in vivo* with L.1 (●) and further exposed to L.1 *in vitro* (○) are reported. Tumour cells from untreated animals were used as −ve controls.

**Figure 2** *In vitro* lysis of L1210 cells treated *in vivo* with L.1. The *in vivo* treatment was performed as reported in Figure 1. L1210 tumour cells collected from L.1 treated mice were labelled with $^{51}$Cr and assayed in the CDC assay in the presence of rabbit complement alone (●) and after further exposure to L.1 *in vitro* (○) (supernatant from culture). Controls were performed using L1210 cells from untreated mice incubated *in vitro* with rabbit complement alone (×) or with L.1 + complement (*).

24 h after the L.1 inoculation and decreased gradually, although 15% of +ve cells were still detected 6 days after the treatment. As shown in Table IV, the amount of L.1 bound to L1210 cells after *in vivo* treatment was dependent upon the dose of the antibody injected. Findings in keeping with those reported in Figure 1 were obtained in the cytotoxic assay. (Figure 2). A % of tumour cells derived from L1210-bearing mice previously treated with L.1 underwent lysis upon *in vitro* incubation with rabbit complement. However, full susceptibility to complement-mediated lysis was obtained upon cell re-exposure to L.1 *in vitro*.

**Table IV** Binding of L.1 to L1210 cells *in vivo*; dose-response relationship

| *L.1*[b] *antibody dilution* | *Fluorescence % stained cells*[a] | *Dye Test %dead cells*[a] |
|---|---|---|
| – | 3±0.1 | 9±0.3 |
| $10^{0}$ | 64±6.9 | 78±0.8 |
| $10^{-1}$ | 67±6.2 | 75±2.5 |
| $10^{-2}$ | 34±7.6 | 33±1.3 |
| $10^{-3}$ | 18±4.1 | 24±0.9 |
| $10^{-4}$ | 6±0.3 | 19±0.1 |

[a]Mean ±s.e..
[b]Tumour-bearing mice were infused i.v. with L.1 (ascites, 0.5 ml/mouse) and, 24 h later, L1210 leukaemic cells were collected and assayed for the presence of L.1 by IMF and by the Dye exclusion test of Gorer & O'Gorman (1956).

*Studies of L.1 properties after* in vivo *administration*

The time course of blood L.1 levels in $CD2F_1$ mice, as evaluated by IMF (dashed line) or by the $^{51}$Cr-release assay (full line), was studied (Figure 3).

**Figure 3** Blood kinetics of L.1 in $CD2F_1$ mice. Serum samples from $CD2F_1$ mice i.v. infused with 1 ml of L.1 (0.2 ml of ascites diluted 1:4) were analyzed for the presence of the MAb in the IMF (●----●) and CDC (●—●) assays at different times after inoculation.

L1210 cells incubated with serum from L.1 treated mice were stained positively in the IMF assay. After 4 days, the binding activity dropped progressively, although it was still detectable 15 days after L.1 inoculation. In sharp contrast, no cytotoxic activity was present in serum from L.1-treated mice. The possibility that complement inactivation by mouse serum was responsible for the *in vivo* loss of L.1 cytotoxic activity, was investigated. Serum samples from $CD2F_1$ mice inoculated i.v. with rabbit complement did not show any complement activity in the CDC (data not shown). Moreover, the presence of undiluted mouse serum in the CDC assay inhibited the cytotoxic reaction, whatever the source of complement (Table V). However, a normal lytic reaction was observed when the mouse serum was removed before the addition of complement.

### *L1210 antigen in mouse fluids*

The detectability of antigen recognized by L.1 shed by tumour cells into the blood or the ascites of L1210-bearing mice was examined. Inhibition of cytotoxic activity of L.1 by serum and ascites fluid from leukaemic mice obtained at varying intervals after the challenge is reported in Figure 4. The inhibitory capacity of mouse fluids, not detectable until 4 days after the tumour inoculation, increased rapidly thereafter and was more impressive for the ascites fluid than for the serum. Titration of soluble antigen (Figure 5) showed that a relatively large amount of free antigen was detectable in the ascites fluid on day 7, whereas a lesser degree of inhibitory activity was demonstrable on day 5.

**Table V** Inhibition of complement activity by normal mouse serum

| *Serum from mouse strain* | *Source of complement** | *cpm ± s.e.* | *% $^{51}Cr$ release* |
|---|---|---|---|
| — | rabbit | 2495 ± 207 | 80.3 |
| $CD2F_1$ | rabbit | 430 ± 38 | 0.2 |
| $CD2F_1$ | rabbit | 426 ± 41 | 0 |
| $CD2F_1$ | rabbit | 448 ± 39 | 0 |
| $CD2F_1$ | guinea pig | 428 ± 46 | 0 |
| $CD2F_1$ | guinea pig | 431 ± 37 | 0.2 |
| $CD2F_1$ | human | 445 ± 48 | 0.8 |
| DBA/2 | rabbit | 412 ± 40 | 0 |
| Balb/c | rabbit | 429 ± 45 | 0 |
| C57B1/6 | rabbit | 410 ± 28 | 0 |
| C3H/f | rabbit | 425 ± 33 | 0 |
| $CD2F_{1(w)}$ | rabbit | 2360 ± 250 | 75.5 |
| $CD2F_{1(w)}$ | guinea pig | 2405 ± 198 | 77.2 |
| $CD2F_{1(w)}$ | human | 2381 ± 279 | 76.3 |

The cytotoxic activity of L.1 was evaluated in a CDC with L1210 target cells, in the presence of normal serum from different strains of mice as described in **Materials and Methods**. When indicated (w), L.1 and mouse serum were washed off before adding the complement. L.1 (supernatant from hybridoma culture) was present in all samples. Replacement of L.1 with rabbit anti mouse serum caused similar results.

*Sources of rabbit complement were selected from non-cytotoxic blood samples from our breeding colony. Guinea pig sources were purchased (Bio Mérieux, France). Human complement was from healthy donors. All samples were assayed *in vitro* for complement activity before use, and for each complement the appropriate dilution was selected. This Table uses rabbit complement as a +ve control.

**Figure 4** Inhibition of the cytotoxic activity of L.1 by ascites and serum from L1210-bearing mice. The supernatant from the hybridoma culture was incubated at 4°C for 45 min with the serum (●—●) and the ascites (●----●) from L1210-bearing mice on different days after the challenge i.p. with $10^4$ leukaemic cells. The mixture was added to L1210-labelled cells and incubated at 37°C (45 min). After washing an appropriate dilution of complement was added as described for the CDC assay. The results are expressed as % inhibition of L.1 cytotoxicity.

### Discussion

In principle, specific and cytotoxic MAb should provide selective bullets for the *in vivo* eradication of cancerous cells leaving normal cells undamaged. These theoretical conditions, hypothesized since the Ehrlich era, were obtained in our laboratory by the production of L.1. In fact, L.1 is absolutely specific to L1210 leukaemic cells and as an IgM immunoglobulin displays great efficiency in fixing complement (Testorelli *et al.*, 1982). Furthermore, since the $CD2F_1$ mice used in these studies were of

**Figure 5** Inhibition of the cytotoxic activity of L.1 by cell-free ascites from L1210-bearing mice. Varying dilutions of cell-free ascites from L1210-bearing mice challenged i.p. with $10^4$ leukaemic cells were incubated at 4°C for 45 min with the supernatant from the hybridoma culture. The residual cytotoxic activity of L.1 was determined in a CDC assay as reported in Figure 4. The inhibitory activity of the ascites obtained on Day 5 (●—●) and on Day 7 (●----●) after the L1210 challenge is illustrated.

identical haplotype to that of the L.1 hybridoma cells, strictly syngeneic conditions could be obtained. However, even under these optimal theoretical conditions, L.1 serotherapy was almost completely ineffective in increasing the survival time of L1210-bearing $CD2F_1$ mice. Variations of the schedule of route or treatment, of the amounts of antibody or of complement were consistently unsuccessful. One reasonable cause for failure, the low level of endogenous complement in mice, was counteracted by excessive inoculation of exogenous complement, but this manoevre was without improvement. Since the activation of an endogenous immune response (Kirch & Hammerling, 1981) is a possible effector mechanism of serotherapy, L.1 might synergize with an effective antitumour drug. Following this line, a number of treatments with cyclophosphamide or with other anticancer compounds (not reported here) were combined with L.1 serotherapy, without any additional increase in the life-span of the mouse.

To account for the failure of L.1 serotherapy several experiments were devised. The first set was conceived to see whether or not L.1 given i.v. to tumour-bearing mice bound to peritoneal L1210 cells. Although the results were positive (Figure 1) a lower percentage than expected of dead cells and of fluorescent cells was regularly observed. It is noteworthy that the fluorescence intensity of *in vivo* L.1-treated cells was 80% below the intensity achieved in the *in vitro*-treated control cells. Decreased susceptibility to *in vitro* complement-mediated lysis by L1210 cells exposed *in vivo* to L.1 was confirmed by the $^{51}Cr$-release assay. Finally, we found that the susceptibility to both complement-mediated lysis and to staining with a second antibody were completely restored after *in vitro* re-exposure to L.1.

The *in vivo* studies suggest the following conclusions: (a) a fraction of L1210 cells did not bind L.1 *in vivo*; (b) the amount of L.1 bound to the surface cells +ve by IMF-was less than for *in vitro*-treated controls, as demonstrated by the lower fluorescence intensity; (c) the *in vivo* L.1 +ve cells, in spite of their low fluorescence intensity, were still susceptible to *in vitro* lysis in presence of an appropriate source of complement; (d) the antigenic specificity defined by L.1 did not undergo modulation, since fluorescence intensity and susceptibility to lysis were fully recovered after *in vitro* re-exposure to L.1: (e) cells −ve by IMF and those resistant to complement-mediated lysis might belong to the same subpopulation.

Although blood clearance of L.1 in the mouse had the half life already described by others for the same antibody class (Bernstein *et al.*, 1980; Kirch & Hammerling 1981), blood serum samples containing L.1 and complement did not lyse L1210 cells *in vitro*. Furthermore, complement from different sources mixed with mouse serum *in vitro* was completely inactivated.

Antigen shedding cannot be responsible for the failure of L.1 serotherapy since, at the start of serotherapy, soluble antigen was not found in body fluids. L.1 inhibition by mouse serum and even more by ascites fluid from tumour-bearing mice became evident 4 days after L1210 challenge. Therefore, circulating antigen might negatively influence late treatments only.

The experimental system used in these studies although adhering to optimal theoretical conditions, did not bring about increased survival time for leukaemic mice. Incomplete antibody binding to target cells and blood inactivation of complement might be major factors in these disappointing results.

A number of claims (Kirch & Hammerling, 1981; Bernstein *et al.*, 1980; Scheinberg & Strand 1982; Miller & Levy, 1981; Ritz *et al.*, 1980; Trowbridge & Lopez 1982; Herlyn & Koprowski, 1981), of therapeutic benefit from the use of MAb in cancer therapy have been reported recently in the literature. Partial successes in tumour systems other than that used here could be attributed to different Ig isotypes, acting through an antibody-dependent cellular cytotoxicity (ADCC) rather than complement-mediated lysis. The limited achievements of this approach might result in greater efforts (Pimm *et al.*, 1982; Raso *et al.*, 1982) to exploit monoclonal technology to carry toxic compounds specifically to their target cells.

Research supported in part by PFCCN, C.N.R., Rome, Italy.

### References

BALL, E.D., KADUSHIN, J.M., SCHACTER, B. & FANGER, M.W. (1982). Studies on the ability of monoclonal antibodies to selectively mediate complement-dependent cytotoxicity of human myelogenous leukemia blast cell. *J. Imminol.*, **128,** 1476.

BERNSTEIN, I.D., TAM, M.R. & NOWINSKI, R.C. (1980). Mouse leukaemia: therapy with monoclonal antibodies against a thymus differentiation antigen. *Science,* **207,** 68.

BERNSTEIN, I.D., NOWINSKI, R.C., TAM, M.R., MCMASTER, B., HOUSTON, L.L. & CLARK, E.A. (1980). Monoclonal antibody therapy of mouse leukaemia. In: *Monoclonal antibodies. Hybridomas: A New Dimension in Biological Analysis.* (Eds. Kennet *et al.*) New York: Plenum Press, p. 275.

GORER, P.A. & O'GORMAN, (1956). The cytotoxic activity of isoantibodies in mice. *Transplant. Bull.*, **3,** 142.

GUNN, B., EMBLETON, M.J. & BALDWIN, R.W. (1981). Monoclonal antibody against a naturally occurring rat mammary carcinoma. *Int. J. Cancer,* **26,** 325.

HERLYN, D.M. & KOPROWSKI, H. (1981). Monoclonal anticolon carcinoma antibodies in complement-dependent cytotoxicity. *Int. J. Cancer,* **27,** 769.

IWAKI, Y., KASAI, M., TERASAKI, P.I. & 7 others. (1982). Monoclonal antibody against $A_1$ Lewis d antigen produced by the hybridoma immunized with a pulmonary carcinoma. *Cancer Res.*, **42,** 409.

KIRCH, M. & HAMMERLING, U. (1981). Immunotherapy of murine leukaemias by monoclonal antibody. Effect of passively administered antibody on growth of transplanted tumor cells. *J. Immunol.*, **127,** 805.

KÖHLER, G. & MILSTEIN, C. (1975). Continuous cultures of fused cells secreting antibody of predefined specificity. *Nature,* **256,** 495.

METZGAR, R.S., BOROWITZ, M.J., JONES N.H. & DOWELL, B.L. (1981). Distribution of common acute lymphoblastic leukaemic antigen in non hematopoietic tissues. *J. Exp. Med.*, **154,** 1249.

MILLER, R.A. & LEVY, R. (1981). Response of cutaneous T cell lymphoma to therapy with hybridoma monoclonal antibody. *Lancet,* **ii,** 226

OZATO, K., MAYER, N. & SACHS, D.H. (1980). Hybridoma cell lines secreting monoclonal antibodies to mouse H-2 and Ia antigens. *J. Immunol.*, **124,** 533.

PIMM, M.V., JONES, J.A., PRICE, M.R., MIDDLE, J.G., EMBLETON, M.J. & BALDWIN, R.W. (1982). Tumour localization of monoclonal antibody against a rat mammary carcinoma and suppression of tumour growth with Adriamycin-antibody conjugates. *Cancer Immunol. Immunother.*, **12,** 125.

RASO, V., RITZ, J., BASALA, M. & SCHLOSSMAN S.F. (1982). Monoclonal antibody-Ricin A chain conjugate selectively cytotoxic for cells bearing the common acute lymphoblastic leukaemia antigen. *Cancer Res.*, **42,** 457.

RITZ, J., PESANDO, J.M., NOTIS-MCCONARTY, J., LAZARUS, H. & SCHLOSSMAN, S.F. (1980). A monoclonal antibody to human acute lymphoblastic leukaemia antigen. *Nature,* **283,** 583.

RITZ, J., PESANDO, J.M., SALLAN, S.E. & 4 others. (1981). Serotherapy of acute lymphoblastic leukaemia with monoclonal antibody. *Blood,* **58,** 141.

SCHEINBERG, D.A. & STRAND, M. (1982). Leukaemic cell targeting and therapy by monoclonal antibody in a mouse model system. *Cancer Res.*, **42,** 44.

SEARS, H.F., HERLYN, D., HERLYN, M., STEPLEWSKI, Z., GROTZINGER, P. & KOPROWSKI, H. (1982). Ex vivo perfusion of human colon with monoclonal anticolorectal cancer antibodies. *Cancer,* **49,** 1231.

TESTORELLI, C., MORELLI, S., GOLDIN, A. & NICOLIN, A. (1982). Characterization of a monoclonal antibody to L1210 leukaemia. *Br. J. Cancer,* **45,** 395.

TROWBRIDGE, I.S. & LOPEZ, F. (1982). Monoclonal antibody to transferin receptor blocks transferrin binding and inhibits human tumor cell growth in vitro. *Proc. Natl Acad. Sci.*, **79,** 1175.

WRIGHT, P.W. & BERNSTEIN, I.D. (1980). Serotherapy of malignant disease. *Prog. Exp. Tumour Res.*, **25,** 140.